# Determinants of anti-vascular action by combretastatin A-4 phosphate: role of nitric oxide

**DOI:** 10.1054/bjoc.2000.1361

**Published:** 2000-08-17

**Authors:** C S Parkins, A L Holder, S A Hill, D J Chaplin, G M Tozer

**Affiliations:** Tumour Microcirculation Group, Gray Laboratory Cancer Research Trust, Mount Vernon Hospital, Northwood, Middlesex, HA6 2JR, UK; Aventis Pharma, Centre de Reserche de Vitry-Alfortville, 13 quai Jules Guesole, BP14, Vitry sur seine, Cedex 94403, France

## Abstract

The anti-vascular action of the tubulin binding agent combretastatin A-4 phosphate (CA-4-P) has been quantified in two types of murine tumour, the breast adenocarcinoma CaNT and the round cell sarcoma SaS. The functional vascular volume, assessed using a fluorescent carbocyanine dye, was significantly reduced at 18 h after CA-4-P treatment in both tumour types, although the degree of reduction was very different in the two tumours. The SaS tumour, which has a higher nitric oxide synthase (NOS) activity than the CaNT tumour, showed ~10-fold greater resistance to vascular damage by CA-4-P. This is consistent with our previous findings, which showed that NO exerts a protective action against this drug. Simultaneous administration of CA-4-P with a NOS inhibitor, N^ω^-nitro-L-arginine (L-NNA), resulted in enhanced vascular damage and cytotoxicity in both tumour types. Administration of diethylamine NO, an NO donor, conferred protection against the vascular damaging effects. Following treatment with CA-4-P, neutrophil infiltration into the tumours, measured by myeloperoxidase (MPO) activity, was significantly increased. Levels of MPO activity also correlated with the levels of vascular injury and cytotoxicity measured in both tumour types. Neutrophilic MPO generates free radicals and may therefore contribute to the vascular damage associated with CA-4-P treatment. MPO activity was significantly increased in the presence of L-NNA, suggesting that the protective effect of NO against CA-4-P-induced vascular injury may be, at least partially, mediated by limiting neutrophil infiltration. The data are consistent with the hypothesis that neutrophil action contributes to vascular injury by CA-4-P and that NO generation acts to protect the tumour vasculature against CA4-P-induced injury. The protective effect of NO is probably associated with an anti-neutrophil action. © 2000 Cancer Research Campaign


British Journal of Cancer (2000) 83(6), 811–816
© 2000 Cancer Research Campaign
doi: 10.1054/ bjoc.2000.1361, available online at http://www.idealibrary.com on IDEAL®

### Determinants of anti-vascular action by combretastatin A-4 phosphate: role of nitric oxide

**CS Parkins[1], AL Holder[1], SA Hill[1], DJ Chaplin[1,2] and GM Tozer[1]**

[1]Tumour Microcirculation Group, Gray Laboratory Cancer Research Trust, Mount Vernon Hospital, Northwood, Middlesex, HA6 2JR, UK; [2]Aventis Pharma, Centre de Reserche de Vitry-Alfortville, 13 quai Jules Guesole, BP14, 94403 Vitry sur seine, Cedex, France

**Summary** The anti-vascular action of the tubulin binding agent combretastatin A-4 phosphate (CA-4-P) has been quantified in two types of murine tumour, the breast adenocarcinoma CaNT and the round cell sarcoma SaS. The functional vascular volume, assessed using a fluorescent carbocyanine dye, was significantly reduced at 18 h after CA-4-P treatment in both tumour types, although the degree of reduction was very different in the two tumours. The SaS tumour, which has a higher nitric oxide synthase (NOS) activity than the CaNT tumour, showed ~10-fold greater resistance to vascular damage by CA-4-P. This is consistent with our previous findings, which showed that NO exerts a protective action against this drug. Simultaneous administration of CA-4-P with a NOS inhibitor, $N^{\omega}$-nitro-L-arginine (L-NNA), resulted in enhanced vascular damage and cytotoxicity in both tumour types. Administration of diethylamine NO, an NO donor, conferred protection against the vascular damaging effects. Following treatment with CA-4-P, neutrophil infiltration into the tumours, measured by myeloperoxidase (MPO) activity, was significantly increased. Levels of MPO activity also correlated with the levels of vascular injury and cytotoxicity measured in both tumour types. Neutrophilic MPO generates free radicals and may therefore contribute to the vascular damage associated with CA-4-P treatment. MPO activity was significantly increased in the presence of L-NNA, suggesting that the protective effect of NO against CA-4-P-induced vascular injury may be, at least partially, mediated by limiting neutrophil infiltration. The data are consistent with the hypothesis that neutrophil action contributes to vascular injury by CA-4-P and that NO generation acts to protect the tumour vasculature against CA4-P-induced injury. The protective effect of NO is probably associated with an anti-neutrophil action. © 2000 Cancer Research Campaign

**Keywords**: combretastatin A-4 phosphate; vasculature; nitric oxide; neutrophil

The identification of tumour vasculature as a potential target in cancer therapy is based on the critical dependence of solid tumour growth upon a functional blood-vessel network (Folkman, 1990). In contrast to anti-angiogenic strategies, anti-vascular targeting aims to cause a rapid and extensive shut-down of the established tumour vasculature, leading to secondary tumour cell death. One of the most promising tumour vascular-targeting drugs is the tubulin-binding agent combretastatin A4-P (CA-4-P), which is currently in Phase I clinical trial. CA-4-P is cleaved to the active but less soluble CA-4 by endogenous non-specific phosphatases. CA-4-P has a high affinity for tubulin at or near the colchicine binding site, thereby inhibiting the polymerization of the tubulin polymers of the cytoskeleton (Pettit et al, 1989; Woods et al, 1995). Systemic administration of CA-4-P causes vascular shut-down, at relatively non-toxic doses, in a range of rodent and human tumours (Chaplin et al, 1996; 1999; Horsman et al, 1998; Grosios et al, 1999). Selectivity to tumour tissue was demonstrated in a rat model (Tozer et al, 1999). Although in vitro studies have demonstrated profound anti-proliferative/cytotoxic and apoptotic effects of CA-4-P against proliferating human umbilical vein endothelial cells (HUVECs) (Iyer et al, 1998; Dark et al, 1997; Grosios et al, 1999), these are only found at relatively high doses.

*Received 31 January 2000*
*Revised 17 May 2000*
*Accepted 19 May 2000*

*Correspondence to:* CS Parkins

The in vivo response of the tumour vasculature is more likely associated with non-cytotoxic effects on endothelial cells, such as condensation of the β-tubulin cytoskeleton and permeability changes, which have been reported for combretastatin A-1 and CA-4-P (Watts et al, 1997; Grosios et al, 1999; S. Galbraith, unpublished data).

Further information is required regarding the mechanism of action of CA-4-P under in vivo conditions. The present study was designed to investigate the role of nitric oxide (NO) in determining the tumour vascular damage induced by CA-4-P. Recent evidence suggests that NO protects against several vascular-damaging strategies. Using two murine tumour types with very different NO production rates, it was found that the high NO-producing tumour was less susceptible to injury induced by oxidative stress following ischaemia-reperfusion (I/R) insult than the low NO-producing tumour (Parkins et al, 1995; 1997). More direct evidence was obtained by nitric oxide synthase (NOS) inhibition, which increased the oxidative stress associated with I/R injury (Parkins et al, 1998) and vascular injury following photodynamic therapy (Korbelik et al, 1997; 2000). Overall, the protective action of NO was observed in a total of six tumour types. Recently, the L-arginine analogue, $N^{\omega}$-nitro-L-arginine (L-NNA) has been shown to potentiate the tumour vascular damage induced by CA-4-P, while having very little effect in a range of normal tissues (Tozer et al, 1999).

Studies of normal tissue inflammation have shown that vascular damage is mediated largely by neutrophil adhesion to the endothelium, with subsequent generation of oxidizing species by neutrophilic myeloperoxidase (MPO) (Grisham et al, 1986; Kettle and Winterbourn, 1997; Kettle et al, 1997; van der Vleit et al, 1997; Eiserich et al, 1998). Nitric oxide strongly attenuates neutrophil-endothelial adhesion (Kubes et al, 1991; Zimmerman et al, 1992) and this pathway is therefore a potential candidate for explaining the protective effect of NO against CA-4-P-induced vascular injury.

In the present study we investigated the role of NO in CA-4-P-induced vascular injury in two types of murine tumour; the CaNT and SaS tumours, which have significantly different NOS activities. Using these tumour models, we tested the hypothesis that the anti-neutrophil actions of NO were responsible for the protective role of NO against CA-4-P-induced vascular damage.

### MATERIALS AND METHODS

### Animals and tumours

Female CBA/Gy f TO mice, aged 12–16 weeks, were used throughout this study. Tumours were implanted in the dorsal subcutaneous region of anaesthetized mice (Metofane, Janssen, Ontario, Canada) with either a crude suspension of the syngeneic breast adenocarcinoma CaNT, containing ~ $1 \times 10^6$ cells, or a 1 $mm^3$ piece of the round cell sarcoma SaS. Tumours were used for experiment when their geometric mean diameter (gmd) reached 6–8 mm (~200–400 mg). The tumour concentration of nitrate, the oxidized form of NO in vivo, is approximately 3 μmol $1^{-1}$ for CaNT and 8 μmol $1^{-1}$ for SaS tumours, as measured by high-performance ion chromatography of microdialysed tumour samples (unpublished data). All animal procedures were carried out under a project licence in accordance with the Home Office (Scientific Procedures) Act, 1986.

### Measurement of vascular volume

The vascular volume of tumours, indicating the volume of perfused blood vessels as a fraction of the volume of tumour tissue was assessed histologically for control and CA-4-P-treated tumours from the perivascular distribution of the fluorescent carbocyanine dye $DiOC_7$ (Molecular Probes, Netherlands) (Trotter et al, 1989). Tumours were assayed at 18 h after treatment with CA-4-P. Briefly, tumours were excised 10 min following a tail vein injection of $DiOC_7$ (0.6 mg $ml^{-1}$ in 75% DMSO in saline, equivalent to 1 mg $kg^{-1}$) and stored frozen at – 20°C. Cryostat sections (10 μm) were prepared and observed using a fluorescence microscope (excitation 480 nm, emission 510 nm). The vascular fraction, i.e. vessels showing perivascular staining by the dye, was estimated by counting a minimum of 100 fields from sections cut at three levels through each tumour, using a morphometric method based on that originally described by Chalkley (Chalkley, 1943; Hill et al, 1995).

### Cell survival excision assay

Measurement of clonogenic cell survival was performed at 18 h after tumour treatment, using an in vivo:in vitro excision assay as previously described (Parkins et al, 1994). Briefly, excised tumours were enzyme-digested to yield viable tumour cells that were plated in culture dishes and incubated until macroscopic colonies were visible and could be counted. Relative cell survival was calculated from the number of clonogens per gram of treated tumour as a fraction of those grown per gram of control tumours.

### Drug treatments

Disodium combretastatin A-4 3-*O*-phosphate (CA-4-P) was kindly provided by Oxigene Corporation (Sweden). The tumour concentration of NO was manipulated by either: inhibition of NO synthase by $N^{\omega}$-nitro-L-arginine (L-NNA) (Sigma, UK) or 1400 W; or supplementation of NO concentration by intravenous injection of diethylamine NO (DEANO) (Molecular Probes, Netherlands), an agent that releases NO upon chemical dissociation. L-NNA and 1400 W were dissolved in saline and administered via intraperitoneal injection at 20 mg $kg^{-1}$ (0.01 ml $g^{-1}$ body weight). 1400 W was kindly provided by Dr LL Thomsen (Glaxo Wellcome Research, Welwyn, Hertfordshire, UK). DEANO, dissolved in 1 mM NaOH, was administered via intravenous injection to achieve a dose of 20 mg $kg^{-1}$. In combination experiments, CA-4-P and L-NNA or DEANO were administered simultaneously.

### Measurement of tumour myeloperoxidase activity

Following treatment by CA-4-P, the degree of neutrophil infiltration was investigated using a modification to a spectrophotometric assay of myeloperoxidase (MPO) (Hotter et al, 1997). Briefly, tumours were homogenized in hexadecyltrimethylammonium bromide in phosphate buffer and after centrifugation the supernatants were assayed for myeloperoxidase activity. Myeloperoxidase activity was assayed spectrophotometrically at 630 nm using the substrate 3,3′,5,5′-tetramethylbenzidine and hydrogen peroxide in phosphate buffer. One unit (U) of enzyme activity is defined as the quantity of protein that produced an increase in absorbance of 1 unit per minute. MPO activity data is presented without correction for the presence of haemoglobin. The total protein content of each homogenate was determined using a commercial kit (Sigma, UK) and enzyme content expressed as U $mg^{-1}$ protein.

### Statistical analysis of data

All the numerical data from analysis of vascular volume, cytotoxicity, neutrophil infiltration and myeloperoxidase activity are expressed as mean value ± 1 standard error of the mean (SEM). Statistical analysis was carried out using Student's t-test (significance achieved if $P < 0.05$).

### RESULTS

### Vascular shutdown studies

Figure 1 shows functional vascular volume measured in the CaNT and SaS tumours at 18 h following treatment with various doses of CA-4-P. The results indicate that significant dose-dependent reductions in functional vascular volume were achieved in both tumour types, with virtually complete shutdown of functional vasculature after 300 mg $kg^{-1}$ dose in CaNT tumours (0.107 ± 0.07% compared to 2.91 ± 0.39% for controls). The SaS tumour vasculature was more resistant to CA-4-P than that of the CaNT with only a partial reduction of functional vascular volume

*© 2000 Cancer Research Campaign*

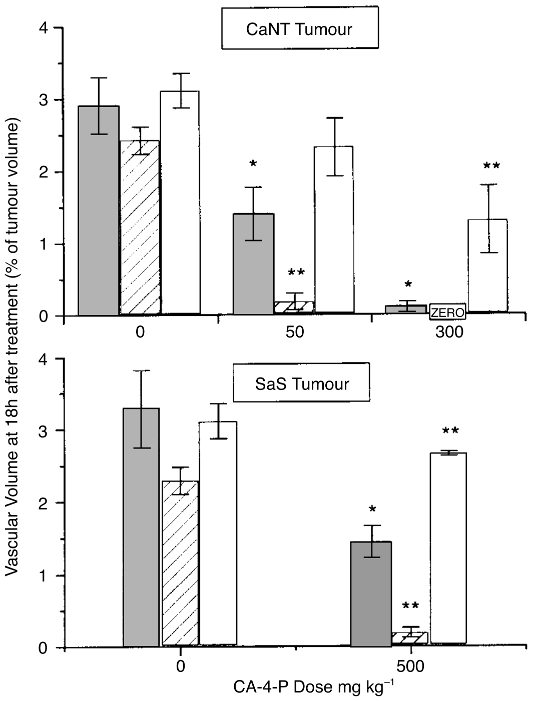

**Figure 1** The functional vascular volume of two types of murine tumour measured 18 h following treatment by a single dose of combretastatin A4-P (CA-4-P). The breast adenocarcinoma CaNT (upper panel) was approximately 10-fold more sensitive to CA-4-P compared to the sarcoma SaS (lower panel) (filled bars). Simultaneous administration of CA-4-P with the NOS inhibitor L-NNA (20 mg $kg^{-1}$) significantly potentiated the vascular damage in both tumour types (hatched bars). A significant protective action against CA-4-P damage was observed by administration of the NO donor DEANO (20 mg $kg^{-1}$), for the highest CA-4-P doses investigated (open bars). Bars are means ± 1 SEM with six tumours per group (* $P < 0.05$ in comparison with controls; ** $P < 0.05$ for enhancement by L-NNA or protection by DEANO)

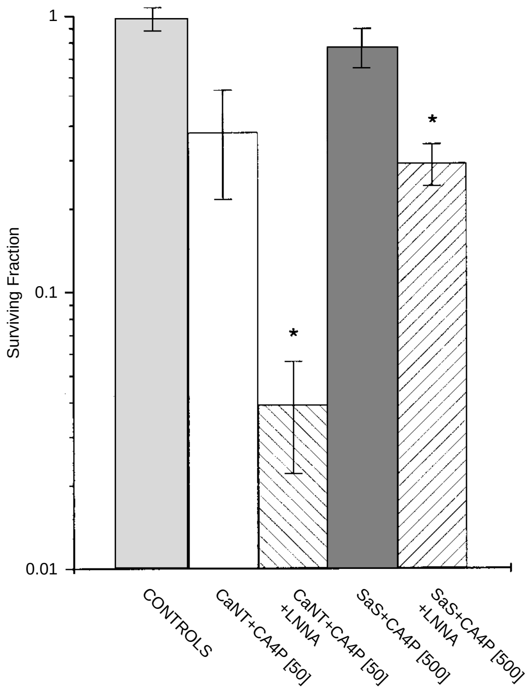

**Figure 2** Clonogenic cell survival was measured in CaNT and SaS tumours at 18 h after treatment with a single dose of CA-4-P, at 50 or 500 mg $kg^{-1}$ respectively, given alone or in combination with L-NNA (20 mg $kg^{-1}$). Tumour cytotoxicity was potentiated in both tumour types by simultaneous administration with L-NNA. The results from both tumour types suggest that intrinsic cellular generation of NO acts to protect against tumour cytotoxicity by CA-4-P. Bars are means ± 1 SEM with six tumours per group (* $P < 0.05$ for enhancement by L-NNA)

following the higher dose of 500 mg $kg^{-1}$ (1.44 ± 0.23% compared to 3.29 ± 0.53% for controls).

Simultaneous administration of the NOS inhibitor L-NNA with CA-4-P resulted in a significantly enhanced shutdown of the vasculature in both tumour types, compared to CA-4-P alone. In the CaNT tumour no functional vessels were visible 18 h after treatment with 300 mg $kg^{-1}$ CA-4-P and L-NNA. The inhibitor alone tended to reduce vascular volume, but this was not significant. Simultaneous administration of CA-4-P with DEANO, an agent that chemically releases NO, resulted in significant protection against the anti-vascular actions of CA-4-P. The protective action of DEANO was observed in both tumour types and the NO donor had no significant effect in the absence of CA-4-P. Administration of the highly selective iNOS inhibitor 1400 W, at 20 mg $kg^{-1}$, did not result in potentiation of the anti-vascular actions of CA-4-P in the SaS tumour (1.57 ± 0.18% compared to 1.44 ± 0.23% for 500 mg $kg^{-1}$ CA-4-P alone) (results not shown).

### Clonogenic cell survival studies

The cytotoxicity associated with a prolonged reduction in tumour vascular volume was assessed using a clonogenic cell survival assay (Figure 2). Tumour cytotoxicity was measured at 18 h following CA-4-P treatment with 50 mg $kg^{-1}$ for CaNT tumour-bearing mice or 500 mg $kg^{-1}$ for SaS tumour-bearing mice. This 10-fold difference in CA-4-P dose was chosen to achieve a similar reduction in functional vascular volume in the two tumours, based on the data shown in Figure 1. Cell survival was significantly reduced in both tumour types when L-NNA was administered simultaneously with CA-4-P, although the effect was much larger for the CaNT tumour. The inhibitor alone did not significantly reduce tumour viability in CaNT or SaS tumours (surviving fraction = 0.57 and 0.78, respectively). A dose-response for CA-4-P-induced cytotoxicity was observed for the CaNT tumour with surviving fraction at 18 h reduced to 0.008 ± 0.006 for a single dose of 300 mg $kg^{-1}$ (results not shown).

### Neutrophil recruitment and myeloperoxidase activities

The degree of neutrophil infiltration into CaNT and SaS tumours was indicated by the total tumour content of neutrophilic myeloperoxidase (MPO), measured at 18 h following CA-4-P treatment (Figure 3). We assume that, at this time, neutrophils were mostly disseminated within the tumour as we have found for I/R injury (unpublished data). The MPO activity for control SaS tumours was approximately 30-fold lower than that for CaNT tumours. Both tumour types showed a CA-4-P dose-dependent increase, although the MPO activity reached remains less in the SaS than in the CaNT tumour. In both tumour types, the simultaneous administration of L-NNA with CA-4-P resulted in an increase in MPO activity compared to CA-4-P alone. This was significant in all cases except for the 500 mg $kg^{-1}$ dose in the SaS

© 2000 Cancer Research Campaign

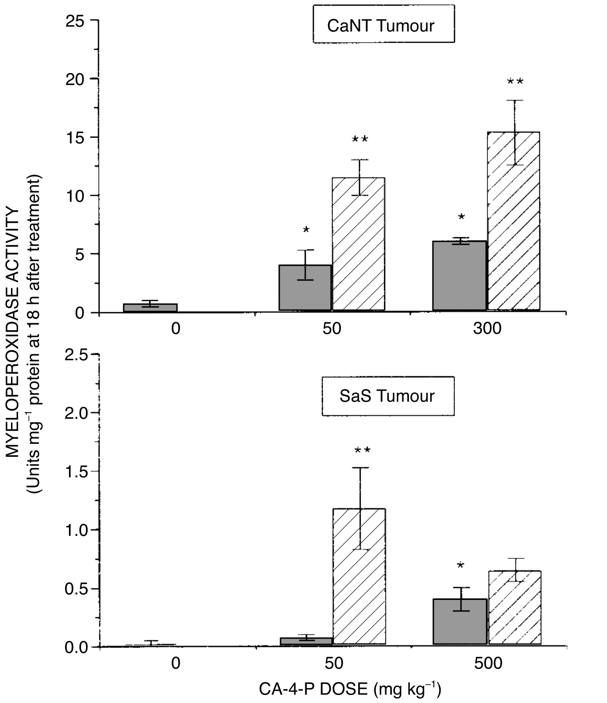

**Figure 3** The degree of neutrophil infiltration induced by CA-4-P treatment of CaNT (upper panel) and SaS tumours (lower panel) was indicated by the total tumour activity of myeloperoxidase (MPO), an enzyme characteristically expressed by neutrophils. A clear CA-4-P dose-dependent increase in MPO activity was shown in both tumour types, although the maximum level reached in SaS tumours was significantly lower than that in CaNT tumours (note the 10-fold difference in the vertical axes) (filled bars). Simultaneous administration of L-NNA (20 mg $kg^{-1}$) with CA-4-P resulted in significant potentiation of neutrophil infiltration in both tumour types with the exception of the 500 mg $kg^{-1}$ dose for the SaS tumour (hatched bars). Bars are means ± 1 SEM with 4–10 tumours per group (*$P < 0.05$ in comparison with controls; ** $P < 0.05$ for enhancement by L-NNA)

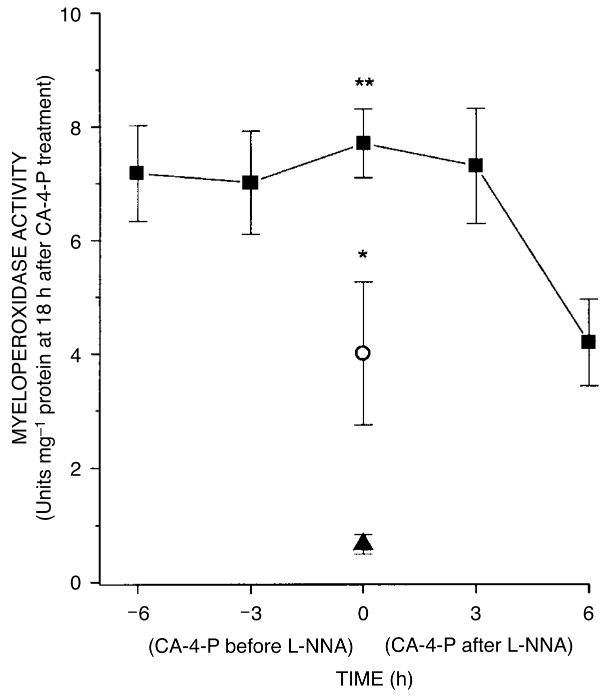

**Figure 4** The time-dependency of administration of CA-4-P with L-NNA (20 mg $kg^{-1}$) was investigated in CaNT tumours by measurement of the total tumour myeloperoxidase content. A similar degree of neutrophil infiltration was observed for all intervals except when CA-4-P was given at 6 h after L-NNA. Symbols: ▲ = control untreated; O = CA-4-P (50) alone; ■ = combinations of CA-4-P and L-NNA (20). Bars are means ± 1 SEM with 4–6 tumours per group (*$P < 0.05$ in comparison with controls; **$P < 0.05$ for enhancement by L-NNA)

tumour. Data for 50 mg $kg^{-1}$ CA-4-P alone indicates an approximately 50-fold difference in MPO activity between the two tumour types, which decreases to approximately 10-fold in the presence of L-NNA. Correction of MPO activity for the presence of haemoglobin in tumour samples did not alter the trends shown by either tumour type.

The potential importance of sequencing the administration of CA-4-P and L-NNA on neutrophil infiltration was investigated in the CaNT tumour (Figure 4). The results indicate that L-NNA given up to 6 h after CA-4-P has a similar effect on neutrophil infiltration as simultaneous administration, although only the simultaneous administration was significantly different from CA-4-P treatment alone. However, pre-treatment with L-NNA is only effective if given within 3 h of CA-4-P treatment.

### DISCUSSION

The present study investigated CA-4-P damage in two different types of murine tumours, previously assessed to have widely differing intrinsic generation of NO. These tumours have previously been characterized for their response to vascular damage following oxidative stress, induced by I/R injury (Parkins et al, 1995; 1997; 1998). The data indicated that NO protects against both neutrophil infiltration and vascular damage following I/R injury, most probably mediated by the inhibitory action of NO against the oxidative stress induced by binding of blood neutrophils to tumour vascular endothelium. In those studies, neutrophil infiltration was quantified by assay of myeloperoxidase activity and found to correlate with the number of infiltrated neutrophils assessed by immuno-histochemical staining. This correlation was evident whether or not the MPO activity was corrected for the presence of haemoglobin (unpublished data). The potentiation of I/R injury evoked by administration of L-NNA was not evident following administration of 1400 W, a potent and selective inhibitor of the inducible isoform of NOS (iNOS). Our current results with CA-4-P are very similar to these, indicating strong parallels between vascular damage evoked by I/R and CA-4-P. In particular, we can say that there is a protective effect of NO production against both types of injury, even in the low NO-producing CaNT tumour, and that this is not due to NO produced by inducible NOS (iNOS). We can therefore hypothesize that NO, produced by constitutive endothelial cell NOS (eNOS), is important in evoking protection against vascular injury induced by both I/R and CA-4-P.

It is evident from the present results that a large part of the protection evoked by NO may be associated with a reduction in neutrophil infiltration. A similar protective role for NO, against neutrophil infiltration, has recently been reported for vascular damage following oxidative stress induced by photodynamic therapy (PDT) (Korbelik et al, 1997; 2000). However, it is not clear from the present study whether the anti-neutrophil actions of NO account entirely for its protective role. A cytoprotective

 © 2000 Cancer Research Campaign effect of NO can occur if partially reduced oxygen intermediates interact with NO and redirect the reactions along a less-damaging pathway (Wink et al, 1993). The vascular effects of CA-4-P occur very rapidly, within minutes of administration (Tozer, unpublished data) and some of the protection evoked by NO may occur before there is any significant CA-4-P-induced neutrophil adherence or infiltration. Although the important roles of NO and neutrophilic myeloperoxidase have been addressed in the present experiments, the observed protective role of NO may be explained, in part, by other published mechanisms. Recent reports have indicated that neutrophilic myeloperoxidase can react directly with nitrate, from NO, to yield nitrotyrosine (Eiserich et al, 1996; 1999). Incorporation of nitrotyrosine into tubulin alters its protein function and has been reported to confer resistance to the cytoskeleton against the action of the tubulin-binding agent, colchicine (Skoufias and Wilson, 1998; Eiserich et al, 1999). The present data on the differences between the two tumour lines would be consistent with this hypothesis if the cytoskeleton of endothelial cells in the SaS tumour contains a higher content of nitrosated proteins than in the CaNT tumour, due to differences in NO concentration. This aspect of nitrotyrosine-induced resistance to tubulin-binding agents, especially CA-4-P, will form part of our future work to identify its role in vascular targeting.

The present results indicate that CA-4-P-induced reduction in vascular volume does not directly correlate with the degree of cytotoxicity measured in the two different tumour types. Although vascular volume was reduced at 18 h following CA-4-P treatment, cytotoxicity will depend upon the extent and duration of vascular shutdown over the whole 18-h period. This time-course may be very different for the two tumour types and cytotoxicity will depend upon the blood-flow remaining in the patent vasculature and changes in oxygen and nutrient consumption of tumour cells following treatment. Our previous studies of I/R injury indicate that recovery of perfusion, following a period of ischaemia, is associated with increased tumour cytotoxicity due to generation of reactive oxygen species from tissue re-oxygenation. Therefore, any differences in the time-course of vascular volume changes between the tumour types would also influence the final cytotoxicity observed. A dose-dependent recovery of blood perfusion has been observed in the rat P22 carcinosarcoma tumour following CA-4-P treatment (Tozer, unpublished data). Significant tumour type-dependent differences in the enzymes xanthine oxidase and dehydrogenase, which are associated with generation of reactive oxygen species upon vascular reperfusion, have been measured in these tumour types and these could influence the cytotoxicity results that we observed following vascular injury by CA-4-P (Anderson et al, 1989; Parkins et al, 1997).

The time-dependence for L-NNA potentiation of CA-4-P-induced neutrophil infiltration appears relatively insensitive for intervals up to 6 h, although significant potentiation was only achieved for simultaneous administration and was lost if CA-4-P was given 6 h after L-NNA. This loss of potentiation was probably due to recovery of NO production at this time. It is likely that L-NNA influences tumour exposure to CA-4-P via a reduction in tumour blood-flow. However, altered pharmacokinetics of CA-4-P are unlikely to explain the current results, as a reduction in flow is likely to decrease tumour exposure to CA-4-P and this is inconsistent with the potentiating effect of L-NNA.

The sensitivity of different tumour types to tubulin-binding agents appears to be, at least partly, determined by mechanisms dependent upon the intrinsic generation and local concentration of NO, combined with factors associated with the tumour content of neutrophilic MPO. The relative importance of these pathways is currently being investigated.

The modifying effects of NO on CA-4-P induced tumour vascular damage may have therapeutic potential. For instance, we have previously shown, in a rat model, that the combination of L-NNA and CA-4-P potentiates vascular damage in the tumour but not in normal tissues (Tozer et al, 1999). It is concluded from the present study that much of this is due to tumour-dependent levels of NO acting to reduce tumour infiltration of neutrophils, thereby reducing damage to the tumour vascular endothelium following CA-4-P. Such an understanding of the role of NO in tumour vascular function will not only be important for the future development of CA-4-P as a cancer chemotherapeutic agent but also for the future development of new vascular targeting agents.

### ACKNOWLEDGEMENTS

We thank Dr Susan Galbraith for permission to quote unpublished data and Oxigene Corporation for supplying the Combretastatin A-4-P. We also thank Gray Laboratory staff for care of the animals and the Cancer Research Campaign and the Gray Laboratory Cancer Research Trust for financial support.

### REFERENCES

Anderson RF, Patel KB, Reghebi K and Hill SA (1989) Conversion of xanthine dehydrogenase to xanthine oxidase as a possible marker for hypoxia in tumours and normal tissues. *Br J Cancer* **60**: 193–197

Chalkley HW (1943) Method for the quantitative morphologic analysis of tissues. *J Natl Cancer Inst* **4**: 47

Chaplin DJ, Petit GR, Parkins CS and Hill SA (1996) Antivascular approaches to solid tumour therapy: evaluation of tubulin binding agents. *Br J Cancer* **74**: S86–S88

Chaplin DJ, Petit GR and Hill SA (1999) Anti-vascular approaches to solid tumour therapy: evaluation of combretastatin A4 phosphate. *Anticancer Res* **19**: 189–196

Dark GG, Hill SA, Prise VE, Tozer GM, Petit GR and Chaplin DJ (1997) Combretastatin A-4, an agent that displays potent and selective toxicity toward tumor vasculature. *Cancer Res* **57**: 1829–1834

Eiserich JP, Cross CE, Jones AD, Halliwell B and van der Vleit A (1996) Formation of nitrating and chlorinating species by reaction of nitrite with hypochlorous acid. *J Biol Chem* **271**: 19199–19208

Eiserich JP, Hristova M, Cross SE, Jones AD, Freeman BA, Halliwell B and van der Vleit A (1998) Formation of nitric oxide derived inflammatory oxidants by myeloperoxidase in neutrophils. *Nature* **391**: 393–397

Eiserich JP, Estevez AG, Bamburg TV, Ye YZ, Chumley PH, Beckman JS and Freeman BA (1999) Microtubule dysfunction by post-translational nitrotyrosination of α-tubulin; A nitric oxide-dependent mechanism of cellular injury. *Proc Natl Acad Sci USA* **96**: 6365–6370

Folkman J (1990) What is the evidence that tumors are angiogenic dependent? *J Natl Cancer Inst* **82**: 4–6

Grisham MB, Hernandez LA and Granger DN (1986) Xanthine oxidase and neutrophil infiltration in intestinal ischaemia. *Am J Physiol* **251**: G567–574

Grosios K, Holwell SE, McGown AT, Pettit GR and Bibby MC (1999) In vivo and in vitro evaluation of combretastatin A-4 and its sodium phosphate prodrug. *Br J Cancer* **81**: 1318–1327

Hill SA, Sampson LE and Chaplin DJ (1995) Antivascular approaches to solid tumour therapy: Evaluation of vinblastine and flavone acetic acid. *Int J Cancer* **63**: 119–123

Horsman MR, Ehrnrooth E, Ladekarl M and Overgaard J (1998) The effect of combretastatin A-4 disodium phosphate in a C3H mouse mammary carcinoma and a variety of murine spontaneous tumors. *Int J Radiat Oncol Biol Phys* **42**: 895–898

© 2000 Cancer Research Campaign

Hotter G, Closa D, Prats N, Pi F, Gelpi E and Rosello-Catafau (1997) Free-radical enhancement promotes leukocyte recruitment through a PAF and LTB4 dependent mechanism. *Free Rad Biol Med* **22**: 947–954

Iyer S, Chaplin DJ, Rosenthal DS, Boulares AH, Li L-Y and Smulson ME (1998) Induction of apoptosis in proliferating human endothelial cells by the tumor-specific anti-angiogenesis agent Combretastatin A-4. *Cancer Res* **58**: 4510–4514

Kettle AJ and Winterbourn CC (1997) Myeloperoxidase: a key regulator of neutrophil oxidant production. *Redox Report* **3**: 3–15

Kettle AJ, van Dalen CJ and Winterbourn CC (1997) Peroxynitrite and myeloperoxidase leave the same footprint in protein nitration. *Redox Report* **3**: 257–258

Korbelik M, Cecic I and Shibuya H (1997) The role of nitric oxide in the response of cancerous lesions to photodynamic therapy. *Recent Res Devel Photochem Photobiol* **1**: 267–276

Korbelik M, Parkins CS, Shibuya H, Cecic I, Stratford MRL and Chaplin DJ (2000) Nitric oxide production by tumour tissue: impact on the response to photodynamic therapy. *Br J Cancer* **82**: 1835–1843

Kubes P, Suzuki M and Granger DN (1991) Nitric oxide: an endogenous modulator of leukocyte adhesion. *Proc Natl Acad Sci USA* **88**: 4651

Parkins CS, Hill SA, Longergan SJ, Horsman MR, Chadwick JA and Chaplin DJ (1994) Ischaemia induced cell death in tumours: importance of temperature and pH. *Int J Radiat Oncol Biol Phys* **29**: 499–503

Parkins CS, Dennis MF, Stratford MRL, Hill SA and Chaplin DJ (1995) Ischaemia-reperfusion injury in solid tumors: the role of oxygen radicals and nitric oxide. *Cancer Res* **55**: 6026–6029

Parkins CS, Hill SA, Stratford MRL, Dennis MF and Chaplin DJ (1997) Metabolic and clonogenic consequences of ischaemia-reperfusion insult in solid tumours. *Exp Physiol* **82**: 361–368

Parkins CS, Holder AL, Dennis MF, Stratford MRL and Chaplin DJ (1998) Involvement of oxygen free radicals in ischaemia-reperfusion injury in murine tumours: role of nitric oxide. *Free Rad Res* **28**: 271–281

Pettit GR, Singh SB, Hamel E, Lin CM, Alberts DS and Garcia-Kendall D (1989) Isolation and structure of the strong cell growth and tubulin inhibitor combretastatin A-4. *Experientia* **45**: 209–211

Skoufias D and Wilson L (1998) Assembly and colchicine binding characteristics of tubulin with maximally tyrosinated and detyrosinated α-tubulins. *Arch Biochem Biophys* **351**: 115–122

Tozer GM, Prise VE, Wilson JI, Locke RJ, Vojnovic B, Stratford MRL, Dennis MF and Chaplin DJ (1999) Combretastatin A-4 phosphate as a tumor vascular-targeting agent: early effects in tumors and normal tissues. *Cancer Res* **59**: 1626–1634

Trotter MJ, Chaplin DJ and Olive PL (1989) Use of a carbocyanine dye as a marker of functional vasculature in murine tumours. *Br J Cancer* **59**: 706–709

van der Vleit A, Eiserich JP, Halliwell B and Cross CE (1997) Formation of reactive nitrogen species during peroxidase-catalysed oxidation of nitrite. *J Biol Chem* **272**: 7617–7625

Watts ME, Woodcock M, Arnold S and Chaplin DJ (1997) Effects of novel and conventional anti-cancer agents on human endothelial permeability: influence of tumour secreted factors. *Anticancer Res* **17**: 71–76

Woods JA, Hadfield JA, Pettit GR, Fox BW and McGown AT (1995) The interaction with tubulin of a series of stilbenes based on combretastatin A-4. *Br J Cancer* **71**: 705–711

Wink DA, Hanbauer I, Krishna MC, DeGraff W, Gamson J and Mitchell JB (1993) Nitric oxide protects against cellular damage and cytotoxicity from reactive oxygen species. *Proc Natl Acad Sci USA* **90**: 9813–9817

Zimmerman BJ, Landreneau FE and Granger DN (1992) The role of neutrophils in reperfusion injury. In: *Molecular basis of oxidative damage by leucocytes*. Jesaitis AJ and Drotz EA (eds), pp 163–169. CRC Press: London

*© 2000 Cancer Research Campaign*
